# RON4_L1_ is a new member of the moving junction complex in *Toxoplasma gondii*

**DOI:** 10.1038/s41598-017-18010-9

**Published:** 2017-12-20

**Authors:** Amandine Guérin, Hiba El Hajj, Diana Penarete-Vargas, Sébastien Besteiro, Maryse Lebrun

**Affiliations:** 1 UMR 5235 CNRS, Université de Montpellier, 34095 Montpellier, France; 2 Department of Internal Medicine and Experimental Pathology, Immunology and Microbiology, American University of Beirut, Beirut, 1107 2020 Lebanon

## Abstract

Apicomplexa parasites, including *Toxoplasma* and *Plasmodium* species, possess a unique invasion mechanism that involves a tight apposition between the parasite and the host plasma membranes, called “moving junction” (MJ). The MJ is formed by the assembly of the microneme protein AMA1, exposed at the surface of the parasite, and the parasite rhoptry neck (RON) protein RON2, exposed at the surface of the host cell. In the host cell, RON2 is associated with three additional parasite RON proteins, RON4, RON5 and RON8. Here we describe RON4_L1_, an additional member of the MJ complex in *Toxoplasma*. RON4_L1_ displays some sequence similarity with RON4 and is cleaved at the C-terminal end before reaching the rhoptry neck. Upon secretion during invasion, RON4_L1_ is associated with MJ and targeted to the cytosolic face of the host membrane. We generated a *RON4*_*L1*_ knock-out cell line and showed that it is not essential for the lytic cycle *in vi*tro, although mutant parasites kill mice less efficiently. Similarly to RON8, RON4_L1_ is a coccidian-specific protein and its traffic to the MJ is not affected in absence of RON2, RON4 and RON5, suggesting the co-existence of independent MJ complexes in tachyzoite of *Toxoplasma*.

## Introduction

Apicomplexa parasites are responsible for important human and animal diseases. The phylum includes significant human pathogens such as *Plasmodium* species responsible for malaria, or *Toxoplasma* the agent of toxoplasmosis. The invasion process is a crucial step for these obligatory intracellular parasites and is mostly conserved throughout the phylum. In most cases, it involves the formation of a unique feature called Moving Junction (MJ). The MJ is a tight apposition between the host cell and parasite plasma membranes. This structure has been first observed in 1978 by electron-microscopy of *Plasmodium knowlesi* merozoites entering red blood cells^1^, then its molecular characterization started being unraveled in *Toxoplasma* tachyzoites (the invasive form of the parasite responsible for the acute phase of the disease) almost 30 years later^2,3^. The molecular components of the MJ are Apicomplexa-specific proteins^2–5^ secreted from two distinct apical organelles of the parasite called micronemes and rhoptries, the latter exhibiting a peculiar club-shape structure with a thin duct (or neck) and a bulbous part^6^. During invasion, the parasite translocates a microneme protein, the apical membrane antigen 1 (AMA1), at its own plasma membrane^7^, and exports a rhoptry neck complex (composed of RON2, RON4, RON5 and RON8 proteins) into the host cell^4^. It should be noted that RON8 is not universally conserved, but seems specific to coccidian parasites such as *Toxoplasma*, *Eimeria* and *Neospora* ^5^. RON2 is inserted into the host plasma membrane and exposes a short segment at the surface of the host cell which interacts with AMA1^8,9^. AMA1 and RON2 thus form an intimate contact^10,11^ which generates a close and irreversible interaction between the parasite and the host cell^12^.

RON4, RON5 and RON8 are soluble proteins, tethered to RON2 and exposed to the cytosolic face of the host cell membrane^4^ where RON2, RON4 and RON5 cooperatively recruit host adaptor proteins that might contribute to anchor the parasite to the host cytoskeleton^13^. Parasites lacking RON4^14^ or RON8^15^ are viable but severely impaired in invasion. In contrast, it has not been possible to generate knock-out mutants for RON2^12^ and RON5^16^, indicating a more critical role during invasion for those proteins. Conditional knock-down (KD) mutants for RON2 and RON5 exhibit an invasion defect of around 90%^12,16^, while KO-RON4 and KO RON8 exhibit 60% and 70% reduction of invasion, respectively. In absence of RON8, the remaining MJ components RON2/RON4/RON5 are correctly targeted to the rhoptries and to the MJ, but 20% of parasites leave trails connecting the PVM with the host cell plasma membrane after invasion^15^. This suggests that RON8 could be required for an efficient fission of the PVM at the end of invasion. In contrast, RON2, RON4 or RON5 depletion induces expression and targeting defects of other members of the MJ complex, resulting in fact in triple functional mutants, hence limiting the phenotypic resolution of individual RON functions^12–14,16^. In KD-RON2, KD-RON5 and KO-RON4 parasites, RON8 correctly localizes to the rhoptries and is found associated with the MJ during invasion in successful invaders^12,13,16^. This indicates that an alternate MJ complex might be formed in absence of RON2, RON4 and RON5 in *Toxoplasma* tachyzoites, also possibly containing additional components. Recent studies showed that a MJ can also be formed in absence of AMA1 in tachyzoites^12,17,18^. Deciphering the architecture of the MJ in complete absence of AMA1 has shown that tachyzoites knock-out for AMA1 upregulate homologs of AMA1 and RON2 that cooperate to support residual invasion^12,19^. Not only this highlighted a likely functional redundancy in MJ components, but it also suggested some variety in MJ proteins, hinting that additional components remained to be discovered. RON4, unlike RON5 and RON8, possesses a putative homolog coded by a gene which was previously named *RON4*_*L1*_^20^ (www.Toxodb.org accession number TGGT1_253370). This gene displays a transcription profile that peaks during the S/M phase, a common characteristic of genes coding for rhoptry proteins^21^. Apart from transcriptional evidence for the *RON4L1* gene^22,23^, nothing was known about the actual localization or role of the corresponding protein in the parasite.

In this report, we further investigate the molecular composition of the MJ complex by characterizing a new rhoptry neck protein, RON4_L1_ that shares sequence homology with RON4 and is highly expressed in tachyzoites. We show that RON4_L1_ is a new member of the MJ complex that is present at the MJ and exposed at the cytosolic face of the host membrane during invasion. We successfully generated a direct knock-out of the *RON4*_*L1*_ gene. RON4_L1_-depleted parasites invade cells similarly as control *in vitro* but are significantly impaired in virulence in mice, a defect restored by complementation with an additional *RON4*_*L1*_ copy. When RON4, RON2 and RON5 are down-regulated, RON4_L1_ expression and its localization in the neck of the rhoptry in intracellular parasites, or at the MJ during invasion, are unchanged. A characteristic ring shape labelling of RON8 and RON4_L1_ is observed in remaining invaders, supporting the existence of an alternate, coccidian-specific, complex for invasion independent of the main and more conserved among Apicomplexa RON2/RON4/RON5 complex. Taken together, our results offer a better understanding of the MJ architecture and support the existence of functional and independent MJ complexes in tachyzoites of *T. gondii*.

## Results

### RON4_L1_ is a new rhoptry neck protein

Transcriptomic and proteomic datasets in ToxoDB indicate that RON4_L1_ is expressed in tachyzoite, bradyzoite and sporozoite stages, as well as in the cat enteroepithelial stage (www.Toxodb.org). Like RON8, RON4_L1_ is a coccidian-specific protein. RON4_L1_ shows 13% identity and 21% similarity with RON4 (Fig. S1): RON4_L1_ is noticeably longer than RON4, and essentially homologous to its C-terminal region. RON4_L1_ is a 1981 amino acids long protein, with a predicted molecular mass of 216 kDa. Besides a predicted signal peptide, no transmembrane domain or any other known recognized domains can be identified.

**Figure 1:**
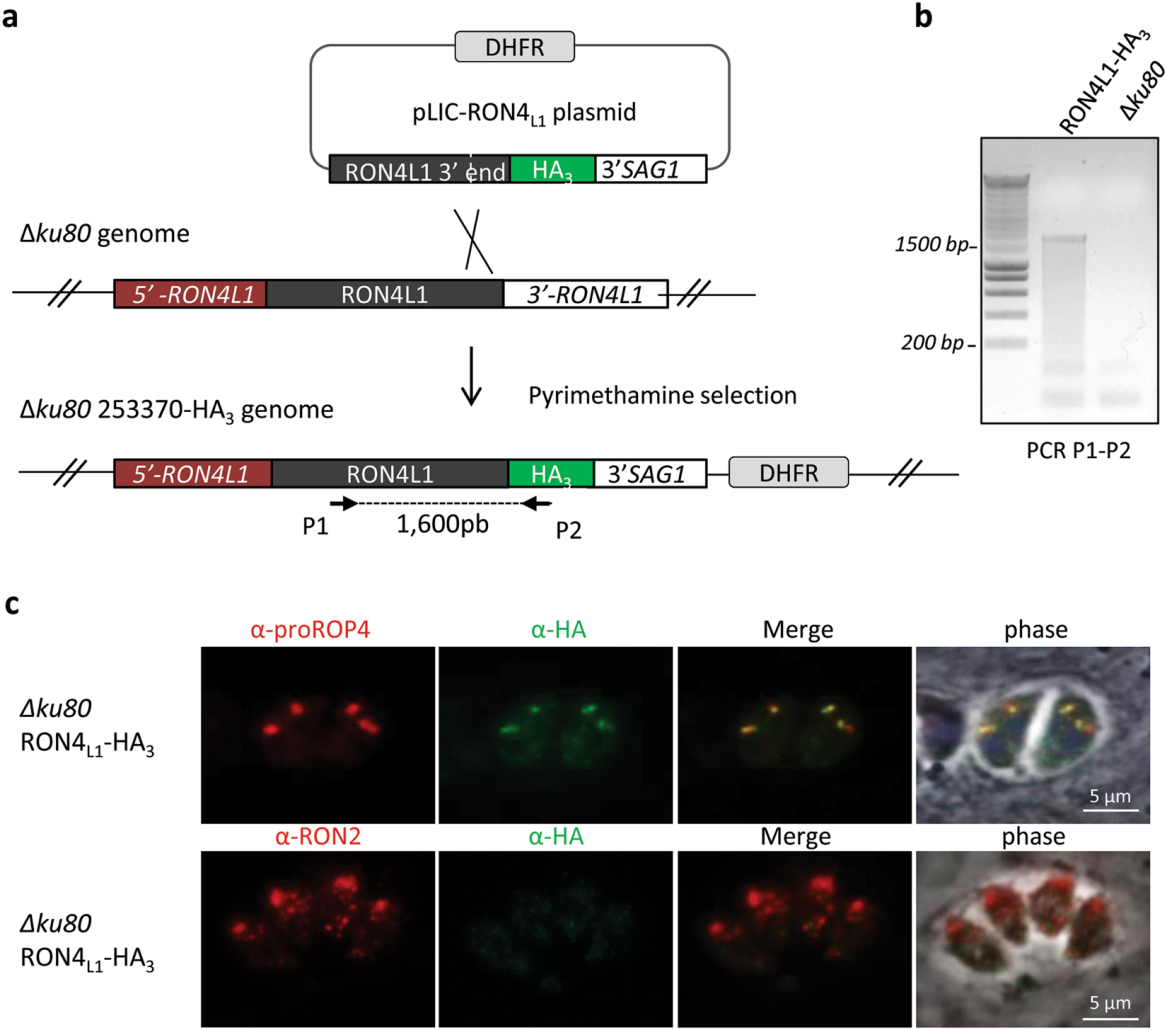
Epitope tagging of RON4_L1_. (**a**) Scheme illustrating the approach used to endogenously tag RON4_L1_ at the C-terminus. Primers used to verify the genetic modification and the size of the PCR product are indicated. (**b**) The HA_3_ tagging at the 3′end of *RON4*_*L1*_ locus by single homologous recombination was verified with PCR using primers P1 and P2, whose positions are indicated in (**a**). (**c**) IFA on intracellular *Δku80* RON4_L1_-HA_3_ parasites using anti-pro-ROP4 and anti-HA antibodies (upper panel) or anti-RON2-4 antibodies (lower panel). The anti-HA antibodies stains only the pre-rhoptries. Scale bars, 5 µm.

**Figure 2:**
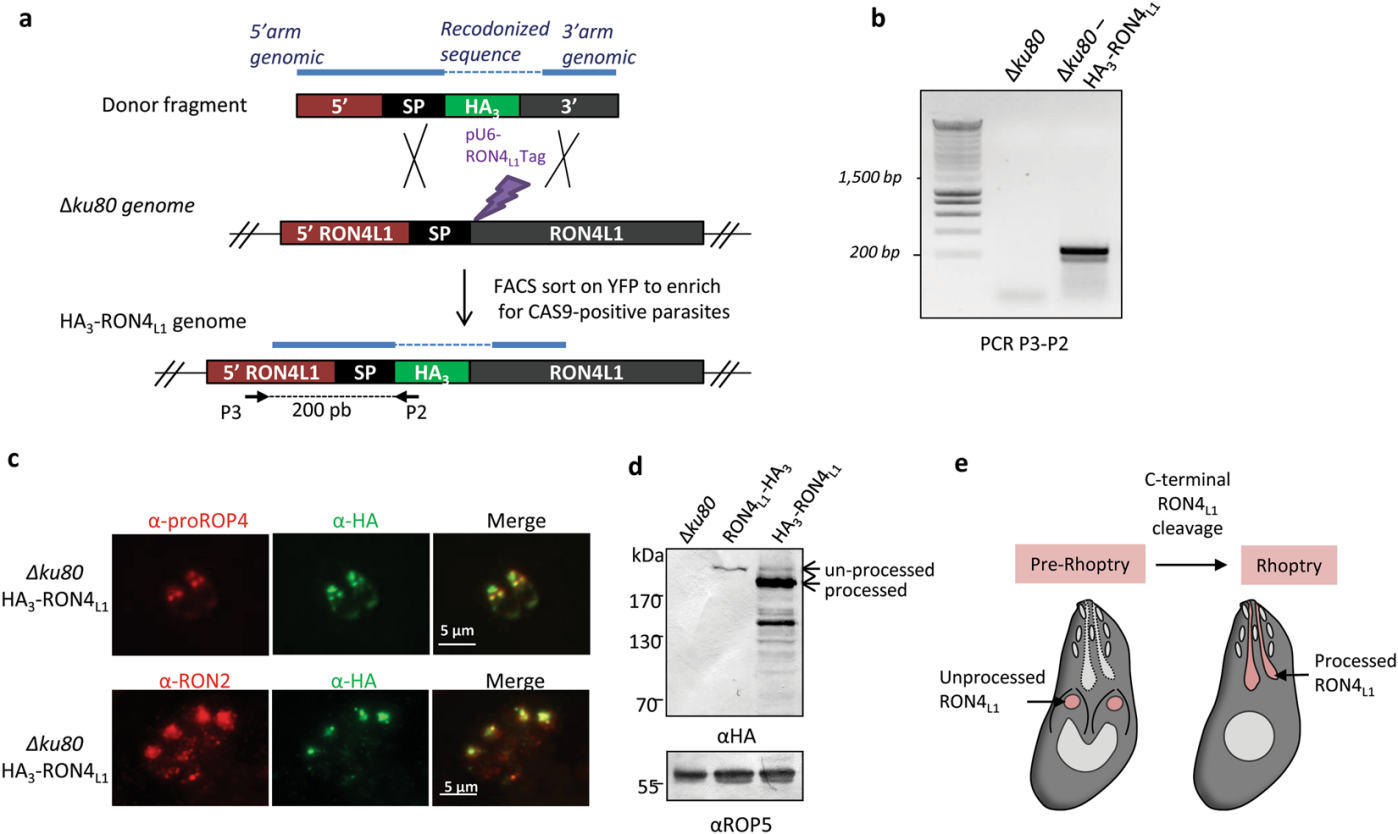
RON4_L1_ is a new rhoptry neck protein, cleaved at the C-terminal end. (**a**) Scheme illustrating the approach used to endogenously tag RON4_L1_ at the N-terminus end in *Δku80* using CRISPR/Cas9 nuclease mediated gene recombination strategy. To induce a double strand break just downstream the sequence signal of RON4_L1_ (black) and introduce a HA_3_-tag by double homologous recombination, a cas9-yfp-RON4_L1_ gRNA expression plasmid (pU6- RON4_L1_ Tag) was transfected together with a linear 442 bp donor DNA fragment containing RON4_L1_ homology regions for double strand break repair. YFP-positive parasites were sorted by FACS to enrich for Cas9-expressing parasites. Recodonized DNA sequence of the donor fragment is hatched. Primers used to verify the genetic modification and the size of the PCR product are indicated. (**b**) The HA_3_ tagging at the 5′end of the coding sequence of *RON4*_*L1*_ locus by double homologous recombination was verified with PCR using primers P3 and P2. (**c**) IFA on intracellular *Δku80* HA_3_-RON4_L1_ parasites using anti-proROP4 and anti-HA antibodies (upper panel) or anti-RON2-4 antibodies (lower panel). The anti-HA antibody stains the pre-rhoptries and the neck of the rhoptry of HA_3_-RON4_L1_ parasites. Scale bars, 5 µm. (**d**) Western blot of *Δku80*, RON4_L1_-HA_3_ and HA_3_-RON4_L1_ strains using anti-HA antibodies. ROP5, loading control. (**e**) Scheme illustrating (on the left) a parasite during endodyogeny process (S/M phase) with immature pre-rhoptries (pink) and (on the right) a parasite in S phase with mature rhoptries. The unprocessed form of RON4_L1_ is present in immature rhoptries while the C-terminal cleaved RON4_L1_ is present in mature rhoptries. Full-length blots/gels are presented in Fig. S6.

To localize RON4_L1_, we first tagged the protein with a triple hemagglutinin tag (HA_3_) at the C-terminal end, by single recombination at the endogenous locus, as described previously^24^ and represented in Fig. 1a. A clone was selected and verified by PCR for proper construct integration (Fig. 1b). IFA using anti-HA antibodies revealed that only a fraction of the parasites display a HA_3_-tag signal, although when present, the signal observed was reminiscent of the pre-rhoptry compartments of developing parasites (data not shown). Because many rhoptry proteins are processed in the secretory pathway in the transition step between immature (pre-rhoptry) and the mature organelle, this labelling suggested that RON4_L1_ is a rhoptry protein potentially undergoing a C-terminal proteolytic maturation. To verify this hypothesis, we first performed co-localization with the pre-rhoptry marker Pro-ROP4^25^. When tagged at its C-terminal-end, RON4_L1_ co-localizes perfectly with the immature ROP4 protein stored in the pre-rhoptries (Fig. 1c, upper panel), while it is absent of mature organelles detected with anti-RON2 antibodies (Fig. 1c, lower panel). This indicated that RON4_L1_ likely travels through the secretory pathway, is addressed to the pre-rhoptries and undergoes a C-terminal cleavage during trafficking to mature organelles. To confirm this hypothesis, we then added a tag at the N-terminus part of the protein in order to follow the mature protein. A strategy was designed to insert a HA_3_-tag after the signal peptide of RON4_L1_ in the *Δku80* RH strain by CRISPR/Cas9-mediated genome editing (Fig. 2a) using a specific donor sequence (Fig. S2) as a template for homologous recombination. Transfected parasites were sorted by FACS to enrich the Cas9-YFP transfected population and then cloned immediately. PCR amplifications (Fig. 2b) and sequencing confirmed the insertion of the HA_3_ tag after the signal peptide of RON4_L1_ in a clone referred hereafter as HA_3_-RON4_L1_, by opposition to the C-terminal tagged RON4_L1_-HA_3_ strain. HA labelling of HA_3_-RON4_L1_ parasites delineated the pre-rhoptries in some parasites, as observed for RON4_L1_-HA_3_ parasites (Fig. 2c upper panel). However, in contrast to the C-terminal tagging, the remaining HA_3_-RON4_L1_ parasites displayed an apical labeling that co-localized perfectly with the rhoptry neck marker RON2 (Fig. 2c lower panel).

Western blot analysis with anti-HA antibody showed that a faint high molecular weight product was observed in protein extracts from the C-terminus tagged cell line, likely corresponding to the unprocessed form (Fig. 2d). While this form was also detected in lysates from the N-terminus tagged cell line, the main product had a lower molecular mass (Fig. 2d). Several lower bands, possibly corresponding to further processing or degradation products, were also detected (Fig. 2d).

In conclusion, RON4_L1_ is a new rhoptry neck protein that undergoes a C-terminal cleavage during rhoptry maturation (Fig. 2e).

### RON4_L1_ is a new component of the MJ RON complex exposed to the cytosolic face of the host cell during invasion

**Figure 3:**
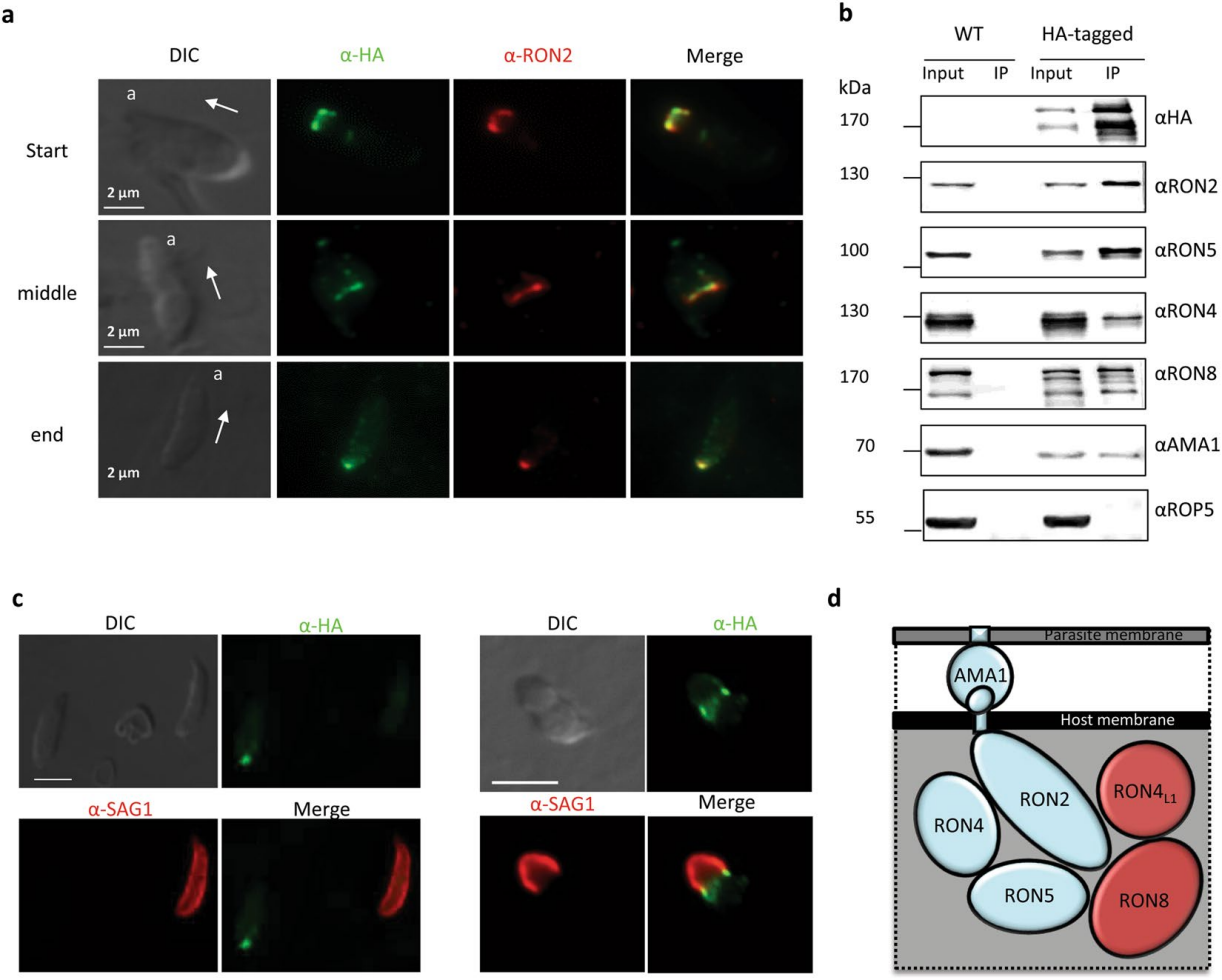
RON4_L1_ is part of the MJ complex AMA1/RON2/RON4/RON5/RON8 and associated with the host cytosolic face of the MJ during invasion. (**a**) IFA on invading *Δku80* HA_3_-RON4_L1_ strain using anti-HA and anti-RON2-4 antibodies. The first two panels illustrate parasites at the beginning and half way of the invasion process. Panel 3 illustrates the staining of RON4_L1_ and RON2 when invasion is complete. RON4_L1_ is detected associated with the MJ in *Δku80* HA_3_-RON4_L1_ parasites. Arrows indicate the direction of motion. a, apical end. Scale bar, 2 µm. DIC: differential interference contrast. (**b**) Co-immunoprecipitation using anti-HA antibodies on *Δku80* HA_3_-RON4_L1_ parasites, followed by an immunoblot using rabbit anti-RON2-3, rat anti-RON8, rabbit anti-RON4, rat anti-RON5, rabbit anti-AMA1 and rat anti-HA antibodies reveals the association of RON4_L1_ with the MJ complex. ROP5, negative control. Full-length blots are presented in Fig. S6. (**c**) Glass beads pre-loading of host cells with anti-HA antibodies to follow the topology of RON4_L1_ at the MJ. Cells were loaded by antibodies directed against HA epitope as described in Materials and Methods and were pulse-infected for 2 min 30 s with *Δku80* HA_3_-RON4_L1_ parasites. The extracellular portion of the tachyzoite was labelled with anti-SAG1 (red), and, then, after permeabilization of the cells with saponin, the host cell-exposed part of the MJ was revealed by addition of the conjugate antibody (green). Image on the left panel shows one parasite intracellular (left) with the residual dot of the MJ labelled with anti-HA antibodies. The parasite on the right is extracellular (labelled with anti-SAG1 antibodies). Image on the right panel shows a parasite in the process of invasion with only half extracellular (red) with the ring of the RON4_L1_ at the MJ (labelled with anti-HA antibodies, green). Scale bar, 5 µm. DIC: differential interference contrast. (**d**) Schematic representation of the current model of MJ complex organization, now including RON4_L1_. AMA1, exposed at the surface of the parasite, interacts with a short portion of RON2, while the rest of the RON2, together with RON4, RON5, RON8 and RON4_L1_ are exposed in the cytosol of the host cell. Conserved RONs proteins in Apicomplexa are in blue, while Coccidian RON8 and RON4_L1_ are in red. Note that the stochiometry of the complex at the MJ is currently unknown.

In order to know if RON4_L1_ is secreted during invasion, we performed IFAs on HA_3_-RON4_L1_ parasites using anti-HA antibodies, with permeabilization conditions optimized to detect only the material secreted by the parasite^26^. Indeed, with low saponin concentration, rhoptries membranes are not permeabilized, and antibodies detect the protein only once secreted from the organelle. On invading parasites, the RON4_L1_ labelling was circumferential, coincided with the constriction of the parasite, and co-localized with the MJ marker RON2 (Fig. 3a, panels 1 and 2). After complete internalization, RON4_L1_ co-localized with RON2 as a puncta systematically observed at the posterior end of the parasite, which corresponds to the residual MJ (Fig. 3a, panel 3). Overall, our results indicate that RON4_L1_ is part of the MJ.

To test whether RON4_L1_ physically interacts with the RON2/RON4/RON5/RON8 MJ complex, we performed an immunoprecipitation using anti-HA beads on a HA_3_-RON4_L1_ parasites lysate. All components of the MJ complex (RON2, RON4, RON5, RON8 and AMA1) were co-immunoprecipitated together with RON4_L1_, even in stringent experimental conditions (1 M NaCl washes) (Fig. 3b), suggesting a strong interaction.

Finally, we sought to test the topology of RON4_L1_ at the MJ. We were unable to identify any putative transmembrane domains in RON4_L1_ using prediction software such as TMHMM (http://www.cbs.dtu.dk/services/TMHMM) or PredictProtein (https://www.predictprotein.org). We thus suspected the protein might be associated with RON4, RON5 and RON8 on the cytoplasmic side of the host cell. To test this possibility, we used the “glass-bead loading” approach previously described to demonstrate the export of RONs and their association with the cytosolic face of the host membrane^4^. The design of this experiment, based on cytoplasmic loading of antibodies within the host cell prior to invasion, allows the detection of RON4_L1_ at the MJ only if the protein is secreted into the host cell cytoplasm (see Methods). This is illustrated by the left panel of Fig. 3c, in which one parasite is extracellular (SAG1 positive, right), while the second is intracellular (SAG1 negative, left) and the latter is also stained with RON4_L1_. This characteristic signal corresponding to the residual MJ shows that RON4_L1_ is exposed on the cytoplasmic side of the host cell. In the same experimental conditions, for invading parasites (Fig. 3c, right panel) RON4_L1_ was also detected at the ring-like MJ.

From these experiments, we conclude that RON4_L1_ is a new member of the MJ complex that is exposed on the cytoplasmic face of the host cell, together with RON2, RON4, RON5 and RON8 (Fig. 3d).

### RON4_L1_ is correctly targeted to the neck of the rhoptry in KD-RON4 and remains associated with the MJ on invading parasites

**Figure 4:**
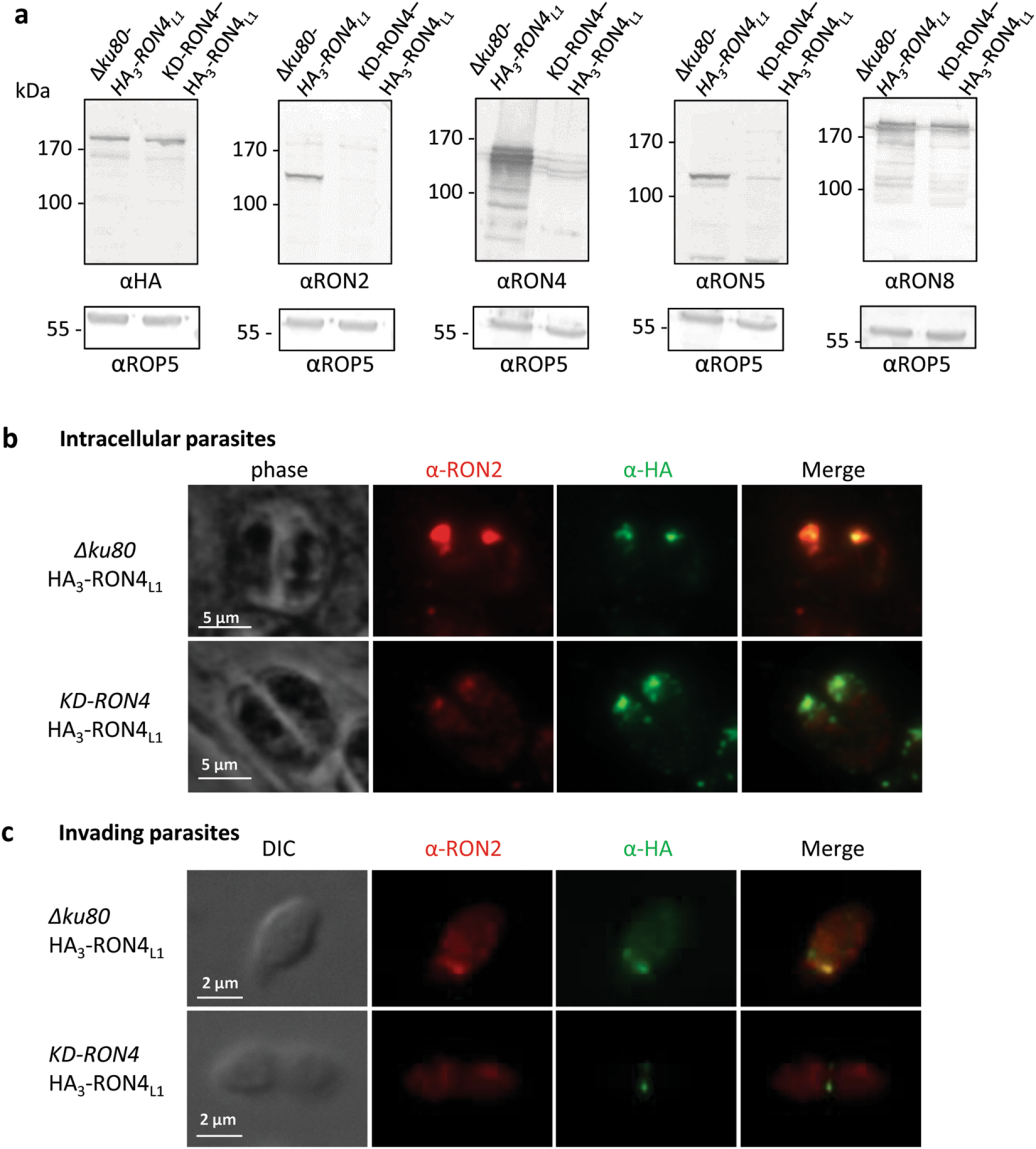
RON4_L1_ expression and localization is independent of the RON2/4/5 complex. (**a**) Western blot analysis of *Δku80* HA_3_-RON4_L1_ and KD-RON4 HA_3_-RON4_L1_ parasite lysates treated with ATc for 48 hours using anti-HA, anti-RON2-3, anti-RON4 rabbit, anti-RON5 and anti-RON8 antibodies. RON4_L1_ expression is not affected by RON2, RON4 and RON5 depletion. ROP5: loading control. Full-length blots are presented in Supplementary Fig. 6. (**b**) IFA of intracellular KD-RON4 parasites in which RON4_L1_ has been HA_3_-tagged. RON4_L1_ localizes in the neck of the rhoptries, as shown by the co-localization with RON2. Scale bar, 2 µm. (**c**) IFA of invading KD-RON4 HA_3_-RON4_L1_ parasites. RON4_L1_ remains present at the moving junction in invading KD-RON4 parasites. Scale bar, 2 µm. DIC: differential interference contrast.

In KD-RON2, KD-RON4 and KD-RON5 mutants, the formation of the RON2/RON4/RON5 complex is disrupted^12,13,16^, while RON8 is expressed in normal amount and traffics correctly to the MJ. We thus sought to investigate the fate of RON4_L1_ in a parasite where the formation of the RON2/RON4/RON5 complex is affected. To this end, we introduced a triple HA epitope-tag after the signal sequence of RON4_L1_ in the KD-RON4 background (Fig. S3), using the same strategy as described in Fig. 2a. As previously observed, altering RON4 expression leads to a significant decrease of RON2 and RON5 expression (Fig. 4a). In contrast, and similarly to RON8, RON4_L1_ is expressed at normal levels (Fig. 4a) and correctly associated with rhoptries in intracellular parasites (Fig. 4b) and at the MJ in invading parasites (Fig. 4c). In conclusion, similarly to RON8, RON4_L1_ traffic seems independent of RON2, RON5 and RON4 proteins expression and complex formation.

### RON4_L1_ plays a role in virulence in a mouse model

**Figure 5:**
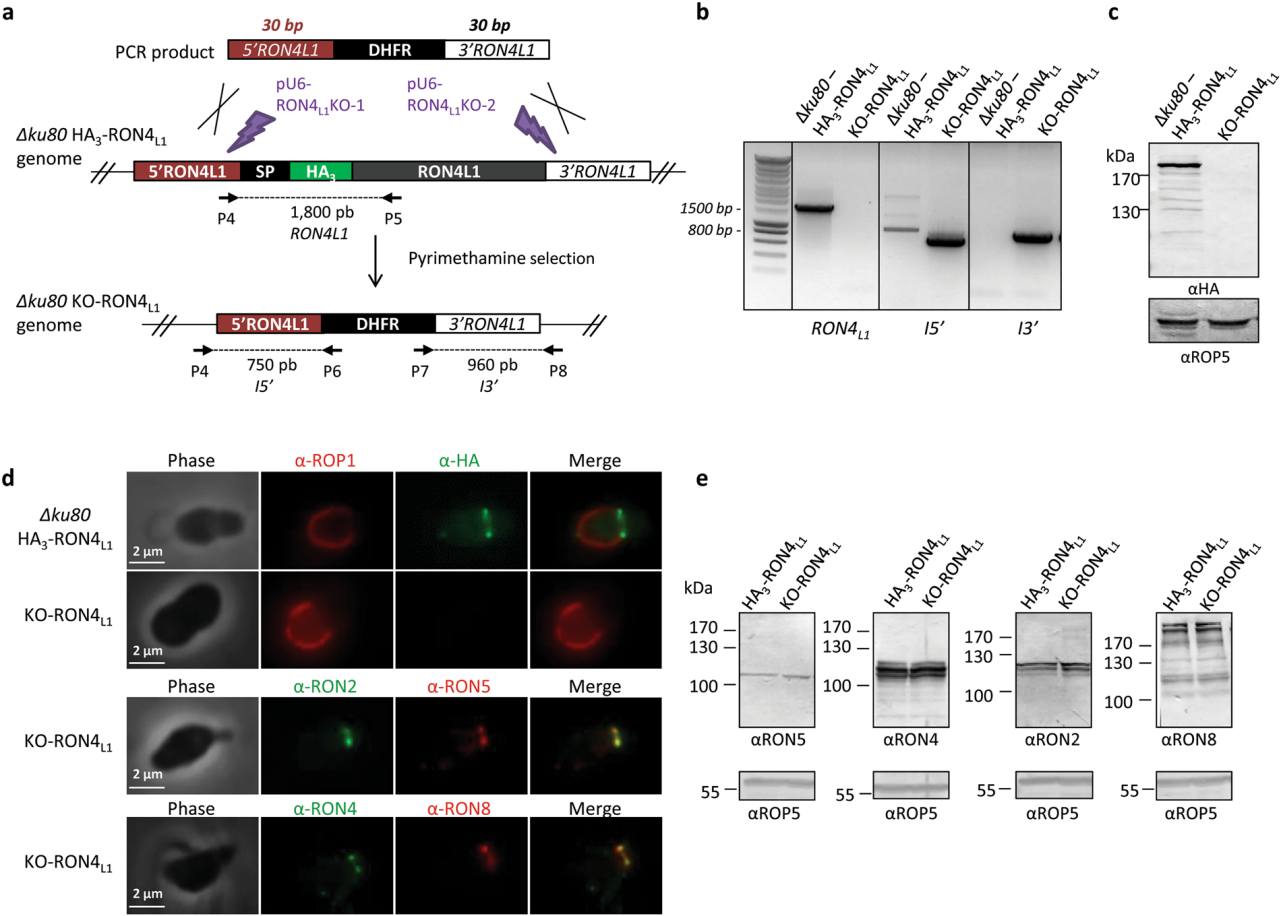
Deletion of RON4_L1_ does not affect the expression and localization of the RON2/RON4/RON5/RON8 complex. (**a**) Scheme representing the approach used to generate a straight knock-out for RON4_L1_ in *Δku80* HA_3_-RON4_L1_ strain. Two protospacers (pU6-RON4_L1_KO-1 and -2; in purple) were designed to induce a double strand break in the 5′UTR and 3′UTR of the *RON4*_*L1*_ gene. A PCR amplifying the DHFR cassette flanked with 30 bp homology sequences of the 5′ and 3′UTR of RON4_L1_ was used as a donor DNA fragment for DNA repair, inducing the removal of the entire *RON4*_*L1*_ gene. Primers used to amplify *RON4*_*L1*_, *I3*′ and *I5*′ fragments and the sizes of the PCR products are indicated. (**b**) PCR verification of DHFR integration at RON4_L1_ locus. (**c**) Western blot using anti-HA antibodies shows the depletion of RON4_L1_ in the KO-RON4_L1_ strain. ROP5: loading control. (**d**) IFA on invading KO-RON4_L1_ parasites using anti-ROP1, anti-HA, anti-RON2-4, anti-RON5, anti-RON4 and anti-RON8 antibodies. RON4_L1_ labelling is lost in KO-RON4_L1_ parasites while the others members of the RON complex are still present at the MJ. Scale bar, 2 µm. (**e**) Western blots analysis using anti-RON2-3, anti-RON5, anti-RON8 and rabbit anti-RON4 antibodies on HA_3_-RON4_L1_ parental line and KO-RON4_L1_ mutant reveal normal expression of RON2, RON4, RON5 and RON8. Full-length blots and gels are presented in Supplementary Fig. 6.

To test the function of RON4_L1_, we used a CRISPR/Cas9 strategy to generate a knock-out cell line by replacing a 22.2 kbp genomic region encompassing the *RON4*_*L1*_ open reading frame by a *DHFR* cassette in the HA_3_-RON4_L1_ cell line background (Fig. 5a). Knock-out parasites (KO-RON4_L1_) were obtained, as verified by diagnostic PCR (Fig. 5b), western blot (Fig. 5c) and IFA (Fig. 5d and Supplementary Fig. S4). This demonstrates RON4_L1_ is not required for parasite survival *in vitro*. While RON4_L1_ is not detected at the MJ of KO-RON4_L1_ parasites, remaining members of the RON complex (RON2, RON4, RON5 and RON8) are still expressed at normal levels (Fig. 5e). The localization of these proteins also remains unaltered: they are present in the neck of the rhoptries in intracellular parasites (Fig. S4) and at the MJ during invasion (Fig. 5d).

**Figure 6:**
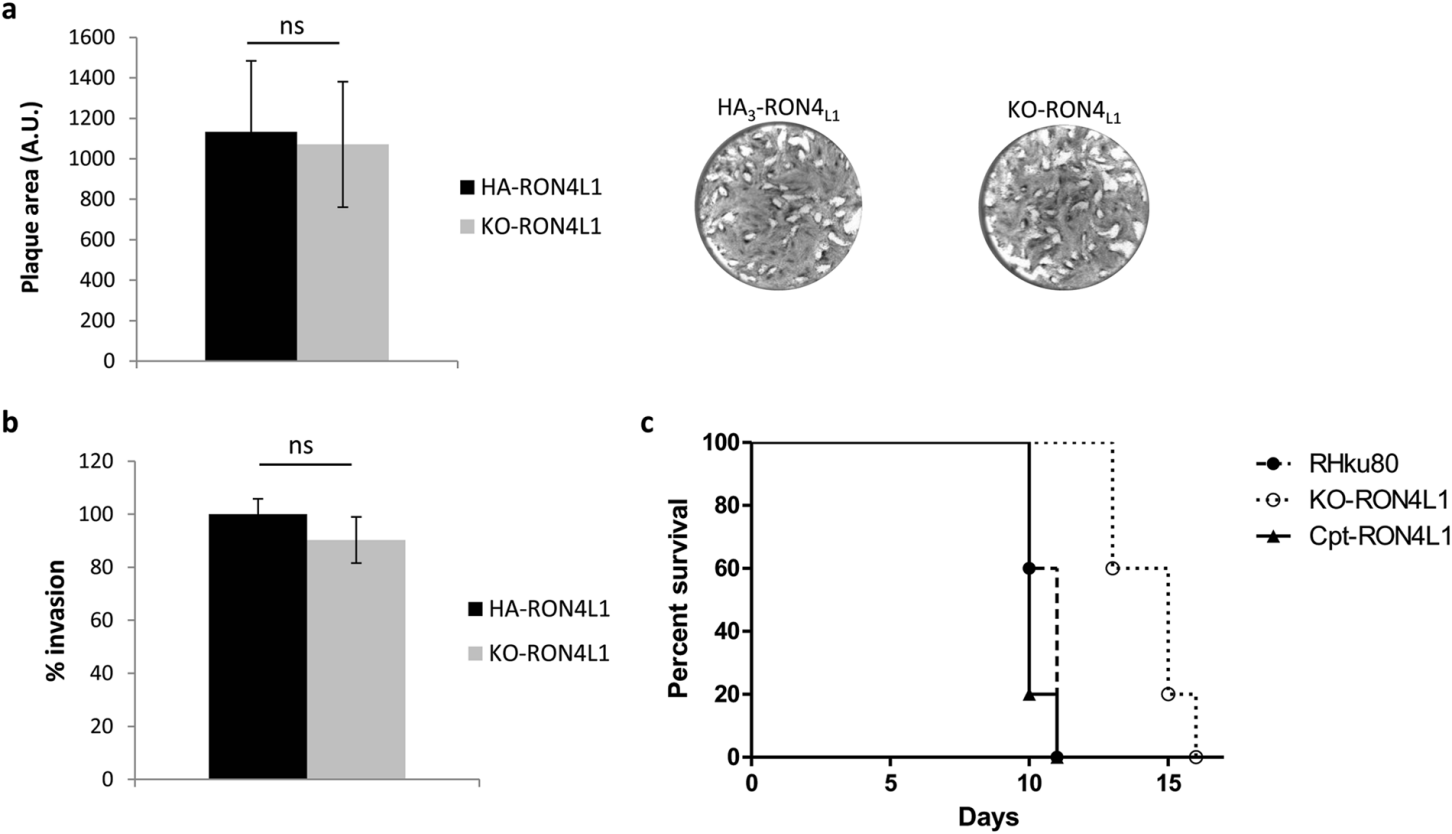
Phenotypic consequences of RON4_L1_ disruption. (**a**) Plaque assay of HA_3_-RON4_L1_ and KO-RON4_L1_ parasites. Parasites were added on HFF monolayer for 7 days and the size of lysis plaques was measured. AU: arbitrary units. Values are the mean standard error of the mean plaque area (20 plaques were measured in each condition) from one representative experiment out of two. No defect of the lytic cycle has been observed in the KO-RON4_L1_ mutant using a two-tailed t-test. (**b**) Invasion assay for 5 minutes in HFF cells of HA_3_-RON4_L1_ and KO-RON4_L1_ parasites. Values represent means ± SD, n = 3, from a representative experiment out of 2 independent assays. No defect of invasion was observed *in vitro* for the KO-RON4_L1_ strain using a two-tailed t-test. (**c**) *In viv*o virulence of *∆ku80*, KO-RON4_L1_ and cpt-RON4_L1_ parasites in a mouse model. n = 5. Logrank tests show a significant difference between WT and KO-RON4_L1_ (P = 0.031) and between KO-RON4_L1_ and Cpt-RON4_L1_ (P = 0.0016), while no difference between WT and Cpt-RON4_L1_ was observed. Representative data out of 2 experiments.

To test the consequence of the loss of RON4_L1_ on the parasite lytic cycle, we then performed a plaque assay, and found that the size of lysis plaques was similar between wild-type and KO-RON4_L1_ parasites (Fig. 6a). Besides, no apparent defect of invasion was detected in fibroblastic cells (Fig. 6b). We next assessed if the loss of RON4_L1_ could impact parasite virulence in the mouse model (Fig. 6c). The mortality of mice infected with KO-RON4_L1_ was delayed (4 to 5 days) compared with that of mice infected with parasites of *Δku80* parental strain or with a cosmid-complemented RON4_L1_-reexpressing cell line (Cpt-RON4_L1_) (Supplementary Fig. S4). These results show that even if the loss of RON4L1 is not significantly impacting *in vitro* growth, the protein contributes to parasite virulence *in vivo*.

## Discussion

The formation of the MJ is an essential mechanism for many Apicomplexan parasites including *Plasmodium* and *Toxoplasma* species. In *Toxoplasma*, the parasite MJ components are composed of AMA1, a micronemal protein exposed on the surface of the parasite, and of a complex of rhoptry neck proteins comprising RON2, RON4, RON5 and RON8. In the present study, we identify a new rhoptry neck protein, RON4_L1_, associated with the MJ complex. RON4_L1_ has been identified through its partial homology with the RON4 protein. In contrast to the paralogs of RON2, RON2_L1_ and RON2_L2_ which are preferentially expressed in sporozoites or bradyzoites, and up-regulated upon depletion of AMA1^12,19,27^, RON4_L1_ is abundant in tachyzoites and is not up-regulated in the KD-RON4 mutant. Thus, RON4_L1_ is a constitutive component of the MJ complex in tachyzoites.

While RON2, RON4 and RON5 are conserved in most Apicomplexa, RON8 and RON4_L1_ are specific to *Coccidia*. Little is known about how RON proteins traffic to the rhoptry neck. RON5 is believed to be important for RON2 stability and RON4 targeting^16^. Similarly, RON2 and RON4 also play a role in stabilizing the RON2/RON4/RON5 complex^8,13,14^. Thus, depletion of either RON2, RON4 or RON5 is sufficient to reduce the expression and localization of the others. In contrast, their depletion does not influence the behavior of the coccidian-specific MJ members RON8^8,13,16^ and RON4_L1_ (this study). Homologs of RON2 and RON4 and RON5 can be found in many Apicomplexa parasites, supporting the idea of a conserved RON2/RON4/RON5 core complex, and the existence of additional members which would be species specific. Both RON8 and RON4_L1_ are soluble proteins exposed to the cytosolic face of the MJ (Fig. 3d). How they associate with the MJ during the progression of invasion in the absence of the core complex remains unknown. This is particularly intriguing, because so far RON2 is the only known membrane anchor for the RON complex in tachyzoites: it is inserted as a transmembrane protein into the host cell surface, and is supposed to serve as a host connector (via AMA1) to the gliding machinery necessary for the progression of internalization^28^. The localization of both RON8 and RON4_L1_ to a MJ-like ring during invasion in absence of RON2 suggests additional RONs might compensate the anchoring function. Whether RON2_L1_, which is normally expressed at very low level in tachyzoite, could be such a candidate, needs further investigations.

While RON2 and RON5 are essential for tachyzoites^12,16^, RON8 and RON4 are dispensable^14,15^. Nevertheless, RON8 depletion led to a 70% reduction of invasion and a failure to close the PVM properly after successful invasion events. This invasion defect translates to radically impaired virulence in infected mice. Here we showed that RON4_L1_ is also not essential *in vitro* and the absence of RON4_L1_ had no visible impact on the formation of the MJ or the vacuole. Accordingly, the *RON4*_*L1*_ knock-out parasites we generated were not affected in the lytic cycle and invaded fibroblast cells as efficiently as wild-type parasites. Nevertheless we cannot exclude a potential compensatory effect through the up-regulation of other(s) gene(s), as it has been observed for RON2 and AMA1^12^. However, interestingly our *in vivo* experiments showed that in absence of RON4_L1_, the parasites were less virulent. A delay of four to five days was observed compared to infection with parental parasites; this small but consistent delay was abolished by complementation with an exogenous *RON4*_*L1*_ copy. It is not yet clear if the attenuation of virulence observed *in vivo* can be directly linked to a defect in invasion that was not discernible in our *in vitro* fibroblast assay. For instance, a specific role for RON4_L1_ during invasion of a peculiar cell type cannot be excluded. In the mouse model, slight alterations in parasite tropism, tissue migration, and invasion success rates can have potential greater consequences on parasite fitness. Transcriptomic and proteomic data sets in ToxoDB show that, in contrast to RON2, RON4, RON5 and RON8, RON4_L1_ is also expressed in the cat enteroepithelial stage. This could suggest also a role for RON4_L1_ in invasion of merozoites in the intestine.

In contrast to other Apicomplexa, Coccidia such as *Toxoplasma* and *Neospora* have very broad host specificity. The expression of additional RON proteins like RON8 and RON4_L1_ might illustrate an enriched MJ proteins repertoire in these parasites that would support their ability to invade a large variety of cell types.

## Methods

### Ethics statement

All animal work was conducted in strict accordance with the AAALAC (Association for Assessment and Accreditation of Laboratory Animal Care International) guidelines and the guide of animal care use book (Guide, NRC 2011). All mice protocols were approved by the Institutional Animal Care and Utilization Committee (IACUC) of the American University of Beirut (IACUC Permit Number IACUC#14-3-295). All animals were housed in specific pathogen-free facilities. Humane endpoints were used as requested by the AUB IACUC according to AAALAC (Association for Assessment and Accreditation of Laboratory Animal Care International) guidelines and guide of animal care use book (Guide, NRC 2011). Mice were sacrificed for any of the following reasons: 1) impaired mobility (the inability to reach food and water); 2) inability to remain upright; 3) clinical dehydration and/or prolonged decreased food intake; 4) weight loss of 15–20%; 5) self-mutilation; 6) lack of grooming behavior/rough/unkempt hair coat for more than 48 hours; 7) significant abdominal distension; 8) unconsciousness with no response to external stimuli. Animals were deeply anesthetized before cervical dislocation. Eye pricks were done following deep anesthesia with isoflurane and all efforts were made to minimize suffering.

### Parasite culture

All *T. gondii* tachyzoites were passaged in human foreskin fibrolasts (HFFs) (American Type Culture Collection-CRL 1634) or Vero cells (American Type culture Collection CCL 81) grown in Dulbecco’s modified essential medium (Gibco-BRL), supplemented with 5% fetal calf serum and 2 mM glutamine. Tachyzoites of the *T. gondii* RH strain deleted for *ku80* gene (*Δku80*)^24^ were used throughout the study.

### Cloning of DNA constructs

Excepted when notified, all PCR amplifications were performed with the Phusion polymerase (NEB Biolabs) and the primers are listed in Supplementary Table S1.

To tag RON4_L1_ at the C terminal end of *Δku80* parasites, we produced a pLIC-RON4_L1_ plasmid based on pLIC-DHFR-HA_3_^24^. The 3′end of *RON4*_*L1*_ gene was amplified with primers P11/P12, cloned in frame with the triple hemagglutinin tag in pLIC-DHFR-HA_3_ vector and linearized by *Xho*I prior to transfection. Single homologous recombination at the endogenous locus allowed the endogenous tagging of RON4_L1_.

To tag RON4_L1_ at the N terminal end just after the signal peptide, we used the CRISPR/Cas9 strategy. We designed a protospacer that would recruits Cas9 to cut 21 bases pairs downstream the end of the sequence signal codon and a donor DNA to insert triple HA by double homologous recombination. The sequence of the donor DNA is provided in Fig. S2. The protospacer sequence GACAATGCCGCCACGTGTGA was cloned by annealing primers P13 and P14 and cloning *Bsa*I site of vector pU6-Cas9-YFP (gift of B. Striepen, UGA). The resulting plasmid pU6- RON4_L1_Tag contains a Cas9-YFP fusion allowing selection of fluorescent parasites by FACS. The template sequence containing the HA_3_ sequence was generated by IDT services and TOPO-cloned. A 442 bases pairs PCR product corresponding to the template was amplified with KOD polymerase (Novagen) using primers P15/P16 and co-tranfected with pU6- RON4_L1_Tag in *Δku80* or KD-RON4 strains.

A CRISPR/Cas9 strategy was settled up to generate a knock-out of the gene *RON4*_*L1*_. A fragment donor corresponding to the DHFR resistance cassette flanked by 30 bp homology arms of *RON4*_*L1*_ gene was amplified with KOD polymerase (Novagen) using primers P17 and P18. Two pU6-Cas9-YFP vectors were constructed. pU6-RON4_L1_KO-1 plasmid contains a protospacer targeting 11 bp after the start codon and pU6-RON4_L1_KO-2 plasmid targets 13 bp before the stop codon. pU6-RON4_L1_KO-1 and 2 plasmids were generated by annealing primers P19 and P20 or P21 and P22, respectively. The PCR product corresponding to the donor fragment and the two pU6-RON4_L1_KO plasmids were co-transfected in *Δku80* HA_3_-RON4_L1_ strain allowing the removal of the entire *RON4*_*L1*_ gDNA of 22.2kbp.

Complementation of the KO-RON4_L1_ has been done through the integration of the cosmid PSBMF65 (ToxoDB), which encompasses the entire gDNA of RON4_L1_ and contains a bleomycin cassette for selection. It was randomly inserted into the genome. Clones were obtained after phleomycin selection.

### Immunoblots

Proteins from freshly egressed tachyzoites were resuspended into SDS buffer, separated on 10% SDS PAGE and transferred to nitrocellulose membranes. Primary antibodies used are listed in Supplementary Table S2 and diluted in 5% non-fat dry milk in TNT buffer (140 mM NaCl, 15 mM Tris, 0.05% Tween20). After three washes with TNT buffer, nitrocellulose membranes were incubated with alkaline phosphatase conjugated secondary antibodies and revealed with 5-bromo-4-chloro-3-indolyl phosphate/nitro blue tetrazolium (BCIP/NBT; Promega).

### Immunofluorescence microscopy

IFAs on intracellular parasites on HFFs cells were conducted as described previously^29^. Note that RON labelling in the rhoptries have been performed after methanol fixation while RON labelling at the moving junction have been performed after formaldehyde 4% fixation, followed by 0, 1% saponin permeabilization. The antibodies used and their dilution for IFA are listed in Supplementary Table S2. Samples were observed with a Zeiss Axioimager epifluorescence microscope. Images were acquired with a Zeiss Axiocam MRm CCD camera driven at the ‘Montpellier resources imagerie’ facility and processed using Zeiss Zen software. Adjustments for brightness and contrast were applied uniformly on the entire image.

### Transfection and selection of transformants

20 × 10^6^ *T. gondii* tachyzoites were transfected by electroporation at 2.02 kV, 50 Ω and 25 μF using an Electro Cell Manipulator 630 (BTX) with 30 μg of plasmid DNA as described previously^30^. 30 µg of CRISPR/Cas9 plasmids plus 5 µg of PCR products using the KOD polymerase were used to transfect parasites. Transgenic parasites were selected by addition of pyrimethamine at 2 μM for pLIC- RON4_L1_ and pKO-RON4_L1_ vectors, and 30 µg/ml of phleomycin for complementation. For N-terminal tagging of RON4_L1_, parasites transiently expressing cas9-YFP-fluorescence were sorted by FACS two days after transfection and cloned into a 96-well plate For each transfection, clones were isolated by limiting dilution cloning and screened by PCR for correct DNA integration.

### Plaque assays and invasion assays

Plaque assays and invasion assays were performed as described previously^31^. Independent invasion assays were performed three times in which at least 20 fields were quantified per coverslip (n = 3).

### Co-immunoprecipitation

Parasites were solubilized in lysis buffer (1% NP40, 50 mM Tris-HCL pH8, 150 mM NaCl, 4 mM EDTA and protease inhibitor) and immunosorption procedures were done using anti-HA antibodies as described previously^12^. After overnight incubation of the lysate on beads, HA-beads are washed 5 times in wash buffer (50 mM Tris pH8, 1 M NaCl and 0.5%NP40). Elution from beads was performed during 5 min at 95 °C with SDS-PAGE sample buffer. Western blots were performed on the eluates using rabbit anti-RON2-3, rabbit anti-RON4, rat anti-RON5, rat anti-RON8, rabbit anti-AMA1 and rat anti-HA antibodies. The antibodies dilutions used for western blot are described in Supplementary Table S2.

### Glass beads antibody loading

Loading of antibodies was performed using glass-beads as originally described^32^. Acid-washed 150–212 µm glass beads (Sigma) were washed 3 times with distilled water. 0.1 mg of beads were then resuspended in 300 µl of the appropriate medium containing commercial anti-HA antibodies (Covalab) diluted at 1/30). HFF cultures growing on coverslips in a 24 wells-plate were washed twice with Hanks’ Balanced Salt Solution (HBSS) before the antibodies-beads solution was put into each well. The beads were rolled onto the coverslip by tilting the plate ∼10 times, until evenly distributed over its surface. The coverslip was then transferred to another well where it was washed 3 times with HBSS and returned to DMEM culture medium and allowed to recover at 37 °C and 5% CO_2_ for 30 minutes. Invasion assays were then carried out by allowing *T. gondii* tachyzoites to sediment on the HFF for 20 minutes at 4 °C and subsequently warming them during 2.5 min at 37 °C to trigger invasion. Invasion was stopped and cells were fixed by adding an excess volume of 4% PAF in HBSS. The extracellular portion of the tachyzoites was labelled with rabbit SAG1. Parasites and cells were then permeabilized with 0.1% saponin and incubated with secondary antibodies.

### Survival *in vivo*

100 tachyzoites freshly harvested from cell culture were inoculated by intra-peritoneal (i.p.) injection in 8–10 week-old female BALB/c mice (Jackson laboratories). Simultaneously to injection, parasites infectivity was evaluated by plaque assay. 7 days post-infection, seropositivity against *Toxoplasma* infection was tested. Survival experiments were done on groups of 5 mice per parasite cell line. Survival experiments were repeated independently twice. Mice survival was checked on a daily basis until their death, endpoint of all experiments. Data were represented as Kaplan and Meier plots using Prism software (Graphpad).

### Statistical analysis

All results are presented as mean values SEM. For invasion and plaque assays, one representative experiment is shown and two-tailed t-test has been used to determine significance. For *in vivo* experiments, levels of significance were determined with the Logrank test using GraphPad.

### Data availability

All relevant data are included in the paper or the Supplementary Information.

## Electronic supplementary material

Supplementary information

## References

[1] Aikawa M, Miller LH, Johnson J, Rabbege J (1978). Erythrocyte entry by malarial parasites. A moving junction between erythrocyte and parasite. J Cell Biol.

[2] Alexander DL, Mital J, Ward GE, Bradley P, Boothroyd JC (2005). Identification of the Moving Junction Complex of *Toxoplasma gondii*: A Collaboration between Distinct Secretory Organelles. PLoS Pathog.

[3] Lebrun M (2005). The rhoptry neck protein RON4 relocalizes at the moving junction during *Toxoplasma gondii* invasion. Cell Microbiol.

[4] Besteiro S, Michelin A, Poncet J, Dubremetz JF, Lebrun M (2009). Export of a *Toxoplasma gondii* rhoptry neck protein complex at the host cell membrane to form the moving junction during invasion. PLoS Pathog.

[5] Straub KW, Cheng SJ, Sohn CS, Bradley PJ (2009). Novel components of the Apicomplexan moving junction reveal conserved and coccidia-restricted elements. Cell Microbiol.

[6] Dubremetz JF (2007). Rhoptries are major players in *Toxoplasma gondii* invasion and host cell interaction. Cell Microbiol.

[7] Narum DL, Thomas AW (1994). Differential localization of full-length and processed forms of PF83/AMA-1 an apical membrane antigen of *Plasmodium falciparum* merozoites. Mol Biochem Parasitol.

[8] Lamarque M (2011). The RON2-AMA1 interaction is a critical step in moving junction-dependent invasion by apicomplexan parasites. PLoS Pathog.

[9] Tyler JS, Boothroyd JC (2011). The C-terminus of *Toxoplasma* RON2 provides the crucial link between AMA1 and the host-associated invasion complex. PLoS Pathog.

[10] Tonkin ML (2011). Host cell invasion by apicomplexan parasites: insights from the co-structure of AMA1 with a RON2 peptide. Science.

[11] Vulliez-Le Normand B (2012). Structural and functional insights into the malaria parasite moving junction complex. PLoS pathogens.

[12] Lamarque MH (2014). Plasticity and redundancy among AMA-RON pairs ensure host cell entry of *Toxoplasma* parasites. Nat Commun.

[13] Guerin A (2017). Efficient invasion by *Toxoplasma* depends on the subversion of host protein networks. Nat Microbiol.

[14] Wang, M. *et al*. The moving junction protein RON4, although not critical, facilitates host cell invasion and stabilizes MJ members. *Parasitology*, 1–8, 10.1017/S0031182017000968 (2017). 10.1017/S0031182017000968 28662729

[15] Straub KW, Peng ED, Hajagos BE, Tyler JS, Bradley PJ (2011). The moving junction protein RON8 facilitates firm attachment and host cell invasion in *Toxoplasma gondii*. PLoS Pathog.

[16] Beck JR, Chen AL, Kim EW, Bradley PJ (2014). RON5 is critical for organization and function of the *Toxoplasma* moving junction complex. PLoS Pathog.

[17] Bargieri DY (2013). Apical membrane antigen 1 mediates apicomplexan parasite attachment but is dispensable for host cell invasion. Nat Commun.

[18] Giovannini D (2011). Independent roles of apical membrane antigen 1 and rhoptry neck proteins during host cell invasion by apicomplexa. Cell Host Microbe.

[19] Parker ML (2016). Dissecting the interface between apicomplexan parasite and host cell: Insights from a divergent AMA-RON2 pair. Proc Natl Acad Sci USA.

[20] Boothroyd JC, Dubremetz JF (2008). Kiss and spit: the dual roles of *Toxoplasma* rhoptries. Nat Rev Microbiol.

[21] Behnke MS (2010). Coordinated progression through two subtranscriptomes underlies the tachyzoite cycle of *Toxoplasma gondii*. PLoS One.

[22] Fritz HM (2012). Transcriptomic analysis of toxoplasma development reveals many novel functions and structures specific to sporozoites and oocysts. PLoS One.

[23] Behnke MS, Zhang TP, Dubey JP, Sibley LD (2014). *Toxoplasma gondii* merozoite gene expression analysis with comparison to the life cycle discloses a unique expression state during enteric development. BMC Genomics.

[24] Huynh MH, Carruthers VB (2009). Tagging of endogenous genes in a *Toxoplasma gondii* strain lacking Ku80. Eukaryot Cell.

[25] Carey KL, Jongco AM, Kim K, Ward GE (2004). The *Toxoplasma gondii* rhoptry protein ROP4 is secreted into the parasitophorous vacuole and becomes phosphorylated in infected cells. Eukaryot Cell.

[26] Carruthers VB, Sibley LD (1997). Sequential protein secretion from three distinct organelles of *Toxoplasma gondii* accompanies invasion of human fibroblasts. Eur J Cell Biol.

[27] Poukchanski A (2013). *Toxoplasma gondii* Sporozoites Invade Host Cells Using Two Novel Paralogues of RON2 and AMA1. PLoS One.

[28] Frenal, K., Dubremetz, J. F., Lebrun, M. & soldati-Favre, D. Gliding motility powers invasion and egress in Apicomplexa. *Nature Reviews* in press (2017). 10.1038/nrmicro.2017.86 28867819

[29] El Hajj H (2008). Molecular signals in the trafficking of *Toxoplasma gondii* protein MIC3 to the micronemes. Eukaryot Cell.

[30] Kim K, Soldati D, Boothroyd JC (1993). Gene replacement in *Toxoplasma gondii* with chloramphenicol acetyltransferase as selectable marker. Science.

[31] Daher W (2015). Lipid kinases are essential for apicoplast homeostasis in *Toxoplasma gondii*. Cell Microbiol.

[32] McNeil PL (1987). & Warder, E. Glass beads load macromolecules into living cells. J Cell Sci.

