# Supplementary material for: RON4L1 is a new member of the moving junction complex in Toxoplasma gondii
Source: Sci Rep. 2017 Dec 20;7:17907. doi: 10.1038/s41598-017-18010-9 (PMC5738351; doi:10.1038/s41598-017-18010-9)
Supplement: Supplementary file 1 — Supplementary information [file 41598_2017_18010_MOESM1_ESM.pdf]

Supplementary Information

RON4$_{L1}$ is a new member of the moving junction complex in *Toxoplasma gondii*

Amandine Guérin[1], Hiba El Hajj[2], Diana Penarete-Vargas[1], Sébastien Besteiro[1], Maryse Lebrun[1]*

[1]UMR 5235 CNRS, Université de Montpellier, 34095 Montpellier, France

[2]Department of Internal Medicine and Experimental Pathology, Immunology and Microbiology, American University of Beirut, Beirut 1107 2020, Lebanon

*Corresponding author: mylebrun@univ-montp2.fr, Phone: + 33 4 67 14 37 45; Fax: + 33 4 67 14 42 86.

Supplementary Figures

Supplementary Figure S1. Alignment of *Toxoplasma* RON4$_{L1}$ amino acid sequence (TGME49_253370) with previously identified RON4 protein (TGME49_229010).

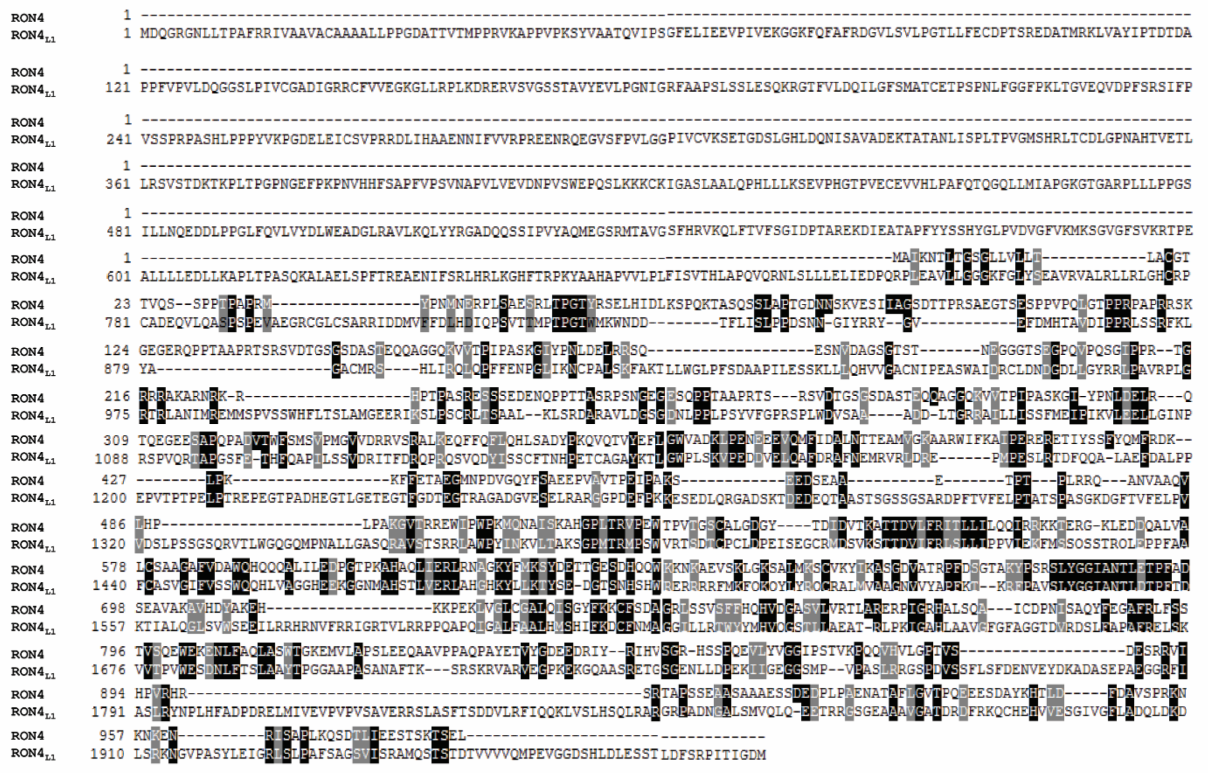

Supplementary Figure S2. **Recodonized sequence used for the N-terminus tagging of RON4$_{L1}$.** Signal peptide in blue, HA_3 tag in green. The recodonized DNA sequence is underlined, the homologous region is in italic and the protospacer in red.

RON4$_{L1}$ donor fragment

AATTCGACCAGCAGTTAGGGAAATTGCAGTCGTTTTCAGATTCAAATGGATCAAGGACGGGGGAATCTCTTGACTCCTGCTT
*TTCGCAGGATCGTGGCGGCTGTAGCTTGTGCTGCAGCCGCCCTTCTGCCACCGGGGGATGCCACA*TACCCGTACGACGTCCC
GGACTACGCTGGCTATCCCTATGATGTCCCAGACTACGCTTACCCATACGACGTCCCAGACTACGCTT**ACTACGGTAACCATGC**
CACCTAGGGTCA*A*AGCTCCGCCGGTCCCTAAATGTAAGATATTGGCGTCTCTCGTTCTTTTTGACTCTTCCGTTTCATCTCGAG*
TGATCCAGTGTTTTGCGCGACGAAAACATTCTAACGAAAACCCAGTTTTGTGTGACACTGGAGCGTTGTAGAGCTGTGCGTC
GTGGCGCCTACCAACAAGCAACAGTTGGG

Signal peptide / HA_3 tag
protospacer / **Recodonized sequence**
In italic: Homologous region

Supplementary Figure S3. Triple HA tagging of $RON4_{L1}$ in the context of the RON4 conditional knock-down mutant.

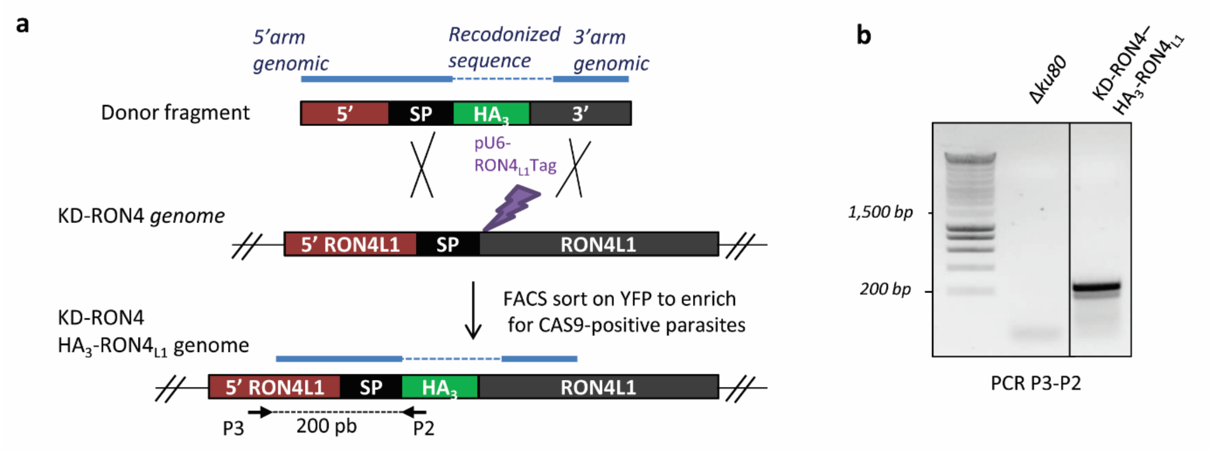

a. Scheme illustrating the approach used to tag endogenous $RON4_{L1}$ at the N-terminus end in *KD-RON4* using CRISPR/Cas9 nuclease-mediated gene recombination. **b.** The HA_3 tagging at the 5'end of the coding sequence of *$RON4_{L1}$* locus by double homologous recombination was verified by PCR using primers P3 and P2.

Supplementary Figure S4. Localisation of RON2, RON4, RON5 and RON8 in absence of RON4$_{L1}$. IFA of intracellular *Δku80* HA$_3$-RON4$_{L1}$ and KO-RON4$_{L1}$ parasites using anti-HA, anti-RON4, anti-RON5, anti-RON2-4 and anti-RON8 antibodies. RON4$_{L1}$ is not detected in the rhoptry neck in the KO-RON4$_{L1}$ strain while RON2, RON4, RON5 and RON8 are stored in the rhoptry compartment. Scale bar, 5µm.

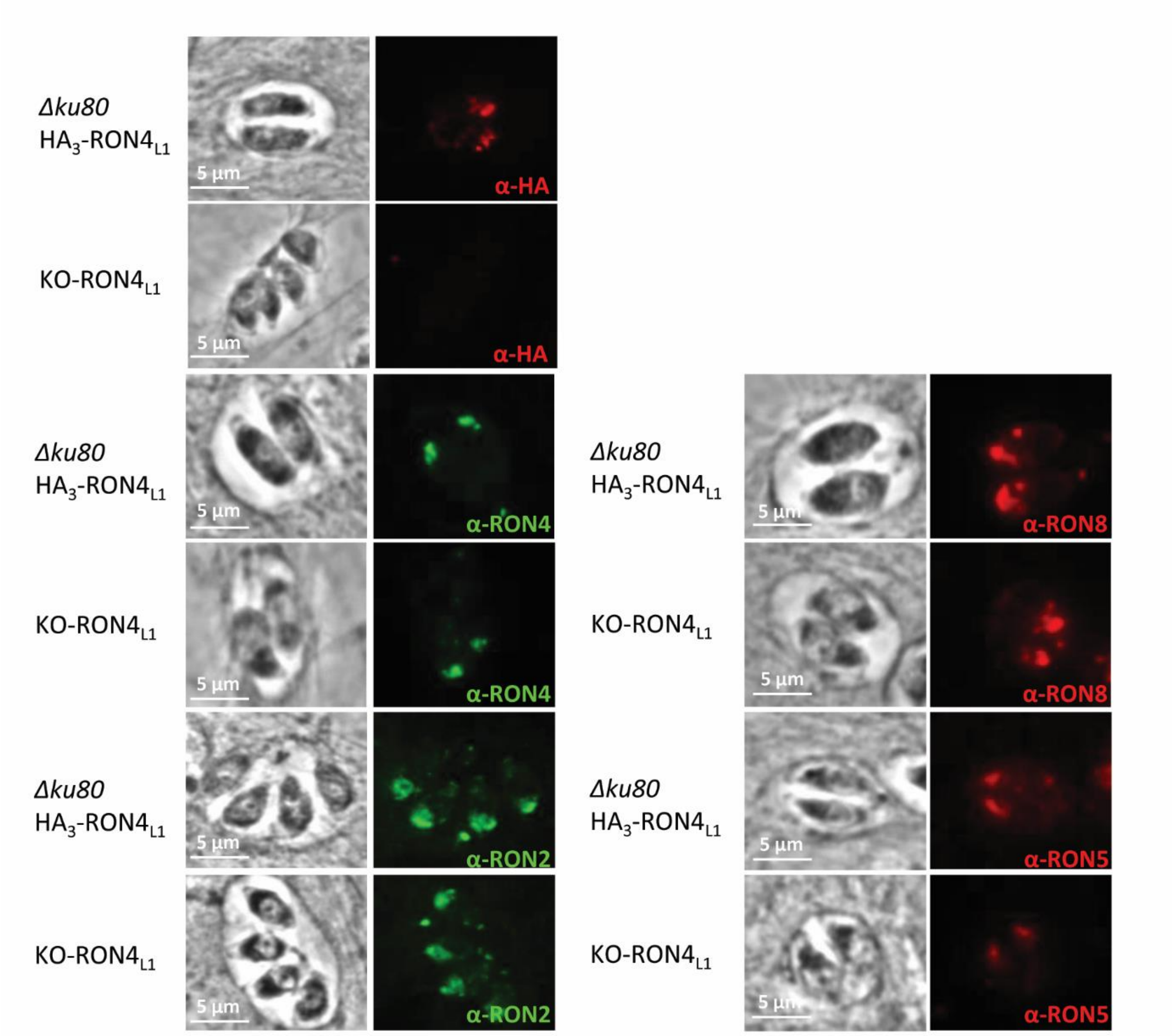

Supplementary Figure S5. Complementation of KO-RON4$_{L1}$ strain.

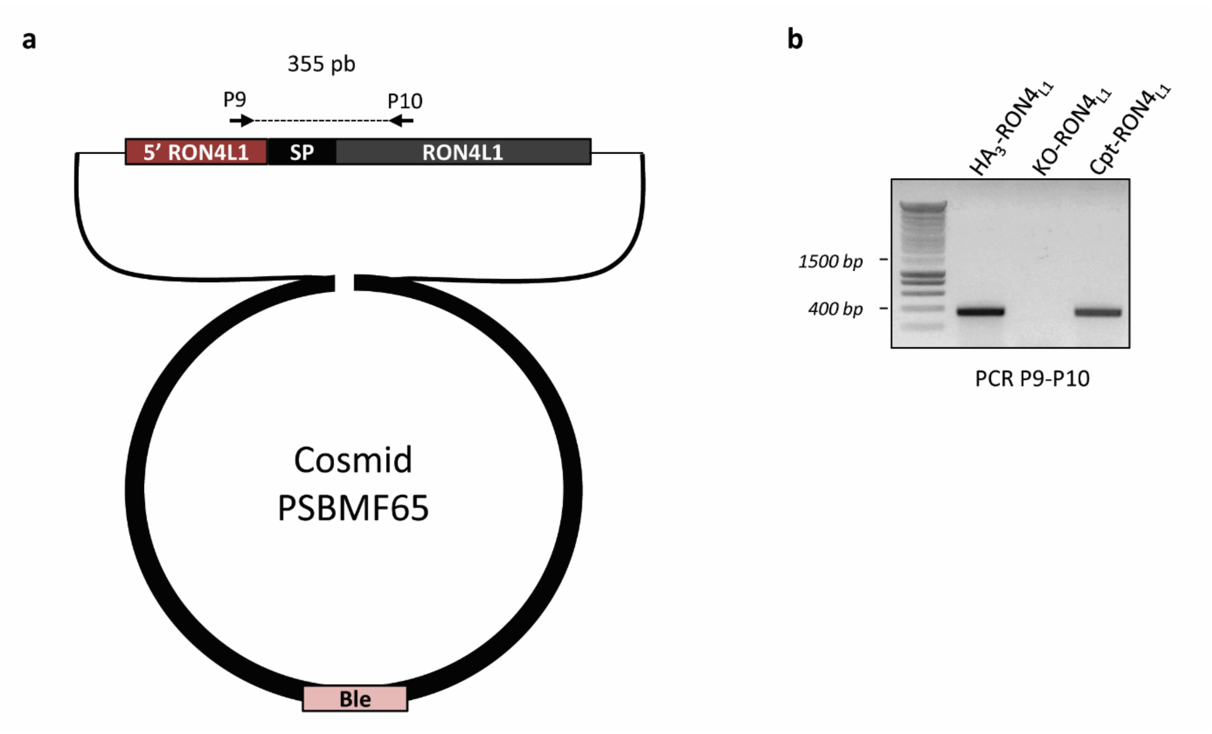

a. Schematic representation of the cosmid PSBMF65 used to complement the KO-RON4$_{L1}$ strain. Primers used to amplify the *RON4$_{L1}$* gene and the size of the PCR product are indicated. **b.** PCR to confirm the presence of the plasmid on the complemented KO-RON4$_{L1}$ strain (Cpt-RON4$_{L1}$).

Supplementary Figure S6. Full length blots and gels

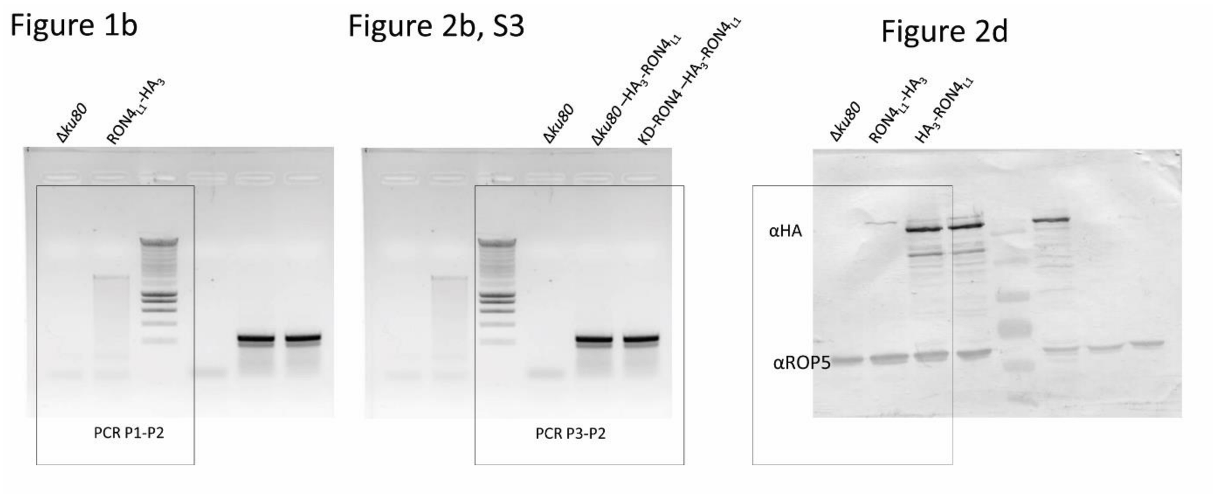

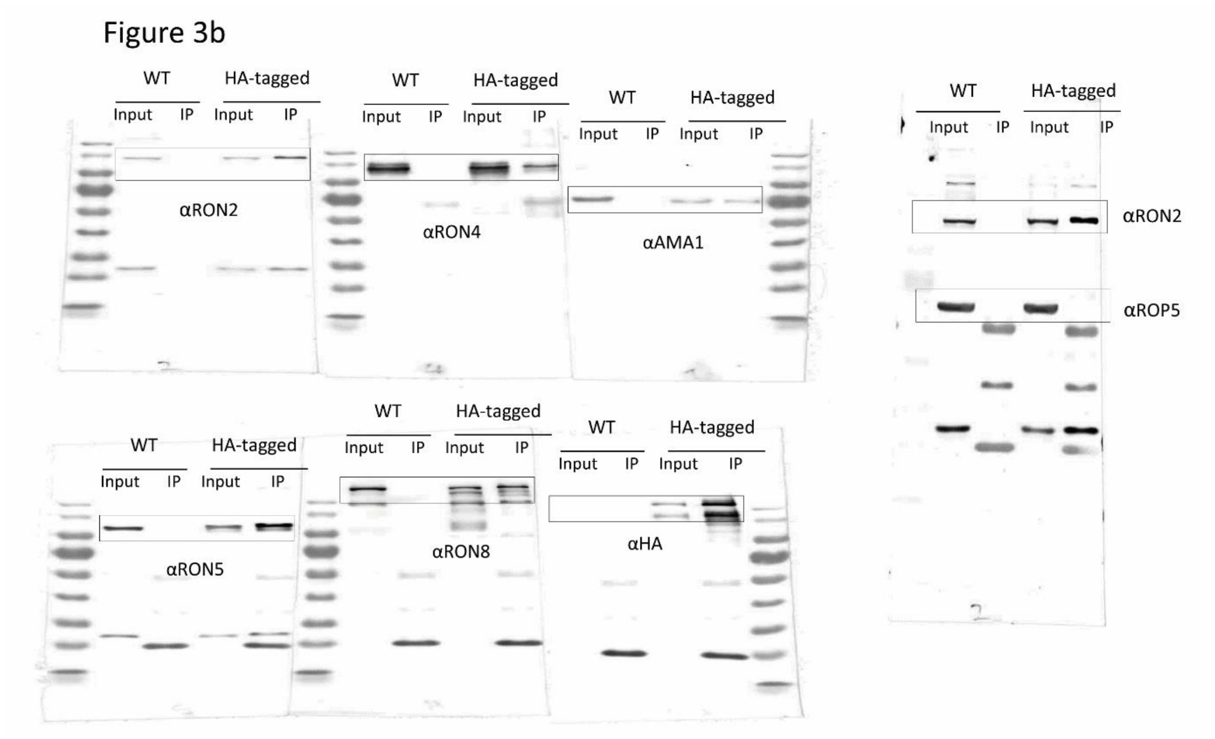

Figure 4a

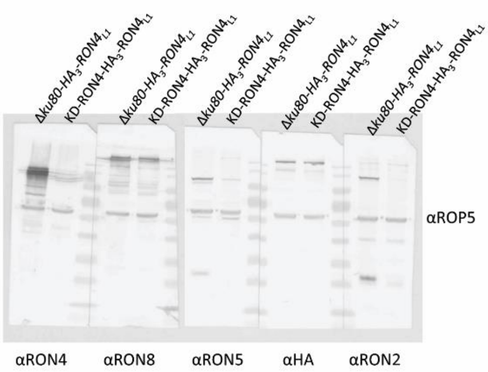

Figure 5b

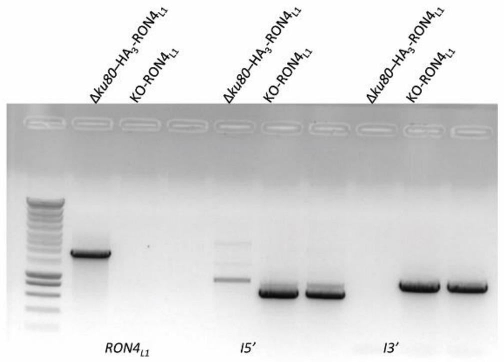

Figure 5c

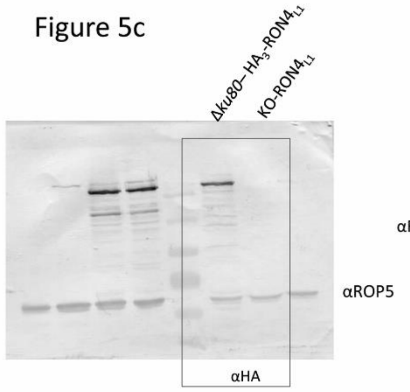

Figure 5e

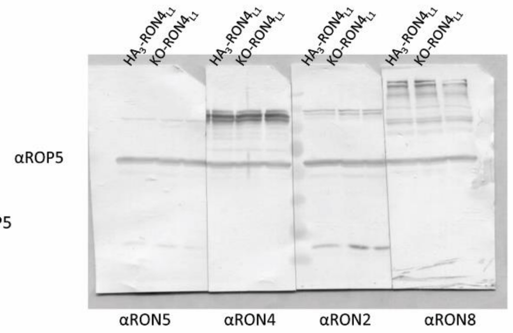

Figure S4b

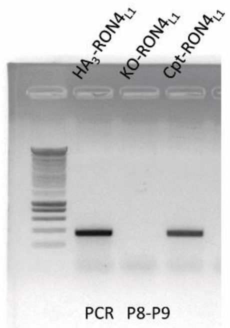

Supplementary Tables

Supplementary Table S1. Primers used.

**P1**	TACTTCCAATCCAATTTAATGCCGGTTCTTTCTCCGTTTTCTTCG
**P2**	CAGCGTAGTCCGGGACGTCGTAC
**P3**	AAGTTGCTGAATTCGACCAGCAGTTAG
**P4**	ACTTCCTCGATCAACTCGAAT
**P5**	AACTATCTAGACGGCGAAGGCA
**P6**	ACGGGAGGCCGTTGTTGAT
**P7**	ACCGAGACCGTTCCCCATT
**P8**	TAGCGAACGTCGGGACTGAA
**P9**	TCTCGCCCGAGCTCTTCGACTT
**P10**	AACGCTCCAGTGTCACACAA
**P11**	CGGGGTACCGCAGCGCGGAGAAGAGGAG
**P12**	TGCATGCATTTGCATCCTCTCGTGAAGT
**P13**	AAGTTGACAATGCCGCCACGTGTGAG
**P14**	AAAACTCACACGTGGCGGCATTGTCA
**P15**	AATTCGACCAGCAGTTAGGG
**P16**	CCCAACTGTTGCTTGTTGGTA
**P17**	TAGGGAAATTGCAGTCGTTTTCAGATTCAAAAGATCCGATCTTGCTGCTG
**P18**	AGGCTTCAGCTTTTTCCTGACTGCAATTCATGGCGGCCGCTCTAGAACTA
**P19**	AAGTTGTTCAAATGGATCAAGGACGGG
**P20**	AAAACCCGTCCTTGATCCATTTGAACA
**P21**	AAGTTGTTCAGTCGTCCAATCACTATG
**P22**	AAAACATAGTGATTGGACGACTGAACA

Supplementary Table S2. Antibodies used.

antibody	Species	WB dilution	IFA dilution	Reference
Anti-RON4	Rabbit	1/1000e	1/1000e	[1]
Anti-SAG1	Mouse<br><br>Mab T4 1E5		1/2000e	[2]
Anti-SAG1	Rabbit	/	1/2000e	[3]
Anti-ROP1 S2b	Rabbit	/	1/1000e	[4]

Anti-RON4	Mouse<br><br>MAb T5 4H1	/	undiluted hybridoma culture	[5]
Anti-ROP5	Mouse<br><br>Mab T5 3E2	1/1000e	/	[5]
Anti-RON2-4	Rabbit	/	1/1000[e]	[6]
Anti-RON2-3	Rabbit	1/1000e	/	[6]
Anti-RON5	Rat	1/500e	1/200e	[4]
Anti-RON8	Rat	1/500[e]	1/200e	[1]
Anti-HA	Rat	1/200[e]	1/100e	Roche, clone 3F10
Anti-proROP4	Rabbit	/	1/1000	[7]
Anti-AMA1 S48	Rabbit	1/5000e	/	[4]

References

1 Besteiro, S., Michelin, A., Poncet, J., Dubremetz, J. F. & Lebrun, M. Export of a *Toxoplasma gondii* rhoptry neck protein complex at the host cell membrane to form the moving junction during invasion. *PLoS Pathog* **5**, e1000309 (2009).

2 Couvreur, G., Sadak, A., Fortier, B. & Dubremetz, J. F. Surface antigens of *Toxoplasma gondii*. *Parasitology* **97 (Pt 1)**, 1-10 (1988).

3 Harning, D., Spenter, J., Metsis, A., Vuust, J. & Petersen, E. Recombinant *Toxoplasma gondii* surface antigen 1 (P30) expressed in *Escherichia coli* is recognized by human *Toxoplasma*-specific immunoglobulin M (IgM) and IgG antibodies. *Clinical and diagnostic laboratory immunology* **3**, 355-357 (1996).

4 Lamarque, M. H. *et al.* Plasticity and redundancy among AMA-RON pairs ensure host cell entry of *Toxoplasma* parasites. *Nat Commun* **5**, 4098, doi:10.1038/ncomms5098 (2014).

5 Leriche, M. A. & Dubremetz, J. F. Characterization of the protein contents of rhoptries and dense granules of *Toxoplasma gondii* tachyzoites by subcellular fractionation and monoclonal antibodies. *Mol Biochem Parasitol* **45**, 249-259 (1991).

6 Lamarque, M. *et al.* The RON2-AMA1 interaction is a critical step in moving junction-dependent invasion by apicomplexan parasites. *PLoS Pathog* **7**, e1001276 (2011).

7 Carey, K. L., Jongco, A. M., Kim, K. & Ward, G. E. The *Toxoplasma gondii* rhoptry protein ROP4 is secreted into the parasitophorous vacuole and becomes phosphorylated in infected cells. *Eukaryot Cell* **3**, 1320-1330 (2004).
